# Identification of key genes and signaling pathways associated with acute pancreatitis and acute lung injury by bioinformatics analysis

**DOI:** 10.1371/journal.pone.0350013

**Published:** 2026-05-22

**Authors:** Lingfeng Chen, Fengzhu Guo, Chunlin Hong

**Affiliations:** 1 Department of Surgery, Zhangzhou Affiliated TCM Hospital of Fujian University of Traditional Chinese Medicine, Zhangzhou, China; 2 Department of Ophthalmology, Zhangzhou Affiliated Hospital of Fujian Medical University, Zhangzhou, China; 3 Department of Hospital Infection Control, Zhangzhou Affiliated TCM Hospital of Fujian University of Traditional Chinese Medicine, Zhangzhou, China; Amity University Noida, INDIA

## Abstract

**Objective:**

To identify key genes and shared pathogenic pathways associated with acute pancreatitis-associated acute lung injury (AP-ALI), through bioinformatics analysis, and to provide potential molecular targets for the diagnosis and treatment of AP-ALI.

**Methods:**

The cerulein-induced severe acute pancreatitis (SAP) mouse lung tissue dataset (GSE244335), lipopolysaccharide (LPS)-induced acute lung injury (ALI) mouse lung tissue dataset (GSE216943), and human AP patient peripheral blood dataset (GSE194331) were retrieved from the Gene Expression Omnibus (GEO) database. Differentially expressed genes (DEGs) were screened. Subsequently, we conducted a series of bioinformatic analyses, including Gene Ontology (GO), Kyoto Encyclopedia of Genes and Genomes (KEGG) pathway analysis, and protein-protein interaction (PPI) network construction. The PPI network was constructed and hub genes were screened. Cross-species consistency of key pathways was verified using the human dataset.

**Results:**

A total of 469 and 153 DEGs were screened from GSE244335 and GSE216943, respectively, with 94 overlapping common DEGs. GO/KEGG enrichment analyses showed that these common DEGs were mainly enriched in immune-inflammatory responses, chemokine receptor binding, and NF-κB signaling pathways. PPI network analysis identified the top 10 hub genes in mice (IL-1β, CCL3, CXCL2, CXCL10, CCL2, CXCL9, CXCL1, CXCR2, TLR2, TNF). Ten hub genes (S100A9, ARG1, RETN, etc.) were screened from the human AP dataset (GSE194331). Cross-species comparison of mouse lung tissue and human peripheral blood revealed 6 common GO-BP terms related to systemic inflammatory responses, suggesting shared mechanisms between local lung injury and systemic inflammation in AP.

**Conclusions:**

The identified hub genes (e.g., IL-1β, CXCL2) and the IL-17/NF-κB signaling pathway represent candidate molecular signatures implicated in AP-ALI. These computational findings generate testable hypotheses regarding inflammatory mechanisms, potentially highlighting shared systemic inflammatory processes between mouse lung injury and human peripheral blood. Direct validation in human pulmonary tissue or bronchoalveolar lavage fluid is warranted to confirm their local pathogenic relevance.

## 1. Introduction

Acute pancreatitis (AP) is an acute pancreatic inflammation, often induced by gallstones or alcohol, characterised by diverse complications driving its severity [1]. Local complications—pancreatic necrosis, pseudocysts, and peripancreatic fluid collections—frequently arise, while systemic responses may progress to sepsis or multi-organ failure [2,3]. These complications elevate morbidity, with severe AP showing 30% mortality, highlighting urgent needs for targeted interventions. Severe acute pancreatitis (SAP) is a life-threatening subtype of pancreatitis, characterized by persistent organ failure and high mortality (~30%), often driven by exaggerated systemic inflammation [4]. A critical complication of severe acute pancreatitis (SAP) is acute lung injury (ALI), which affects 40–60% of patients and arises from a complex interplay of proinflammatory cytokines and neutrophil-driven alveolar damage [5].

ALI associated with AP (AP-ALI) has a complex pathogenesis, and previous studies have shown that it involves a coordinated network of inflammatory mediators and signalling pathways. TLR2 activation by pancreatic damage-associated molecular patterns promotes TNF and IL-1β release via NF-κB signalling, as demonstrated in experimental AP models [5]. These cytokines upregulate CXCL1, which recruits neutrophils through CXCR2 binding, enhancing pulmonary neutrophil infiltration [6]. CCL2 amplifies inflammation by attracting monocytes via CCR1, as observed in neutrophil depletion studies where CCL2-CCR1 blockade reduced monocyte infiltration in ALI models [7]. Neutrophils adhere to alveolar endothelium via Itgam (CD11b) and release MMP9, disrupting alveolar-capillary barriers and exacerbating ALI [8]. These interrelated pathways reveal certain potential therapeutic targets for AP-ALI. However, the key signaling pathways driving AP-ALI pathogenesis remain incompletely understood, necessitating further exploration of potential hub genes and functional proteins.

Nowadays, bioinformatics analyses have emerged as dynamic tools for uncovering potential molecular mechanisms underlying diverse diseases [9]. Yet, a systematic and comprehensive bioinformatics analysis targeting AP-ALI is still lacking. To explore the pathogenic mechanisms of AP-ALI, we retrieved an AP dataset and an ALI dataset from the Gene Expression Omnibus (GEO) database, a gene expression repository maintained by the National Center for Biotechnology Information (NCBI, available at: https://www.ncbi.nlm.nih.gov/geo/). Using multiple integrated bioinformatics tools, we identified hub genes associated with AP-ALI and predicted shared molecular pathogenic mechanisms. We hypothesized that LPS-induced ALI shares conserved molecular mechanisms with AP-ALI regarding neutrophil infiltration and inflammatory signaling, despite different etiologies. This integrative design aims to distinguish pan-inflammatory responses from pancreatitis-specific pathways, thereby identifying transferable therapeutic targets.

## 2. Materials and methods

### 2.1. Data collection

In the present study, datasets were retrieved from the Gene Expression Omnibus (GEO), a gene expression repository maintained by the National Center for Biotechnology Information (NCBI, available at https://www.ncbi.nlm.nih.gov/geo/). The GEO datasets employed in this study included preprocessed and normalized data, rendering them suitable for direct use in downstream analyses. A dataset of SAP mice modeled with cerulein (GSE244335), and a dataset of ALI mice modeled with Lipopolysaccharide (LPS) (GSE216943), were used. GSE244335 contains 3 cases of SAP mice lung tissues and 3 cases of normal mice lung tissues. GSE216943 contains 6 cases of ALI mice lung tissues and 6 cases of normal mice lung tissues. In addition, a dataset on AP patients (GSE194331) was used to validate the analysis results. GSE194331 includes peripheral blood samples from 87 AP patients and 32 healthy individuals.

In the context of this retrospective study, the datasets utilized were retrieved for research-related purposes on July 22, 2025. With respect to participant confidentiality, no information enabling the identification of individual study participants was accessible to the authors at any stage—either during the data collection process or following its conclusion. Meanwhile, due to participant confidentiality and the public sharing of the data, this study neither required nor was able to obtain participant consent.

### 2.2. Analysis of differentially expressed genes (DEGs)

R (version 4.3.3; https://www.r-project.org/) was employed for data processing and standardization. The downloaded files were handled using the “limma” package in R, with subsequent steps including calibration, normalization, log_2_ transformation, and identifing differences between the experimental and normal mice gene expression. The threshold for differential analysis was set at an adjusted p-value (Benjamini-Hochberg false discovery rate, FDR) < 0.05 and a |log_2_ FC| > 2. Volcano plots were generated using web-based tool Science and Research online plot (SRplot; https://www.bioinformatics.com.cn/srplot). The online Venn diagram tool (https://bioinformatics.psb.ugent.be/webtools/Venn/) was used to obtain common DEGs by taking the intersection of DEGs from the two datasets.

### 2.3. Enrichment analysis for the DEGs

Functional annotation based on Gene Ontology (GO) and pathway enrichment analysis using the Kyoto Encyclopedia of Genes and Genomes (KEGG) were conducted via the Database for Annotation, Visualization and Integrated Discovery (DAVID; https://david.ncifcrf.gov/). GO functional annotation was analyzed from three aspects: biological process (BP), cellular component (CC), and molecular function (MF). A significance threshold of adjusted p-value < 0.05 was applied for the analyses.The results of GO and KEGG analyses were imported into SRplot, and bubble plots were drawn to visualize the results.

### 2.4. Generation of protein-protein interaction (PPI) network and hub gene selection

A protein-protein interaction (PPI) network related to AP-ALI was constructed using the Search Tool for the Retrieval of Interacting Genes/Proteins (STRING; http://stringdb.org/) at a medium confidence threshold of 0.400. The PPI networks obtained from the analysis were visualized and further analyzed using Cytoscape (https://cytoscape.org/), a robust bioinformatics tool. Within Cytoscape, the top 10 hub genes were identified using the maximum cluster centrality (MCC) function of the CytoHubba plugin. The selection of the top 10 hub genes follows standard practice in network pharmacology studies using CytoHubba [10]. For example, similar studies analyzing inflammatory networks consistently select the top 5–20 genes by MCC scores for downstream validation [11].

### 2.5. Enrichment analysis for the hub genes

The hub genes screened by Cytoscape were further functionally annotated via GO and underwent KEGG-based pathway enrichment analysis, with both analyses performed using DAVID. GO functional annotation covered three dimensions: biological process (BP), cellular component (CC), and molecular function (MF). An adjusted p-value < 0.05 was set as the significance threshold. The results of GO and KEGG analyses were imported into SRplot to generate bubble plots.

### 2.6. Validation of the analysis results

The DEGs between AP patients and healthy individuals in GSE194331 were screened using the “limma” package in R (the threshold for differential analysis set at an adjusted p-value < 0.05 and a |log_2_FC| > 2). These DEGs were imported into STRING to construct an AP-related PPI network with a medium confidence threshold of 0.400. The PPI network was then imported into Cytoscape software, and the MCC function of the CytoHubba plugin was used to screen out 10 hub genes associated with the occurrence and development of human AP. The hub genes underwent functional annotation based on GO, with the DAVID tool employed for this process. Up to this point, both the GO-BP pathways of the common hub genes in GSE244335 and GSE216943, as well as the GO-BP pathways of the hub genes in GSE194331, have been clarified. The online Venn diagram tool was further used to find the intersection, aiming to identify whether there are overlapping parts between these two types of GO-BP pathways. Due to ethical constraints limiting access to human AP-ALI lung tissue, we utilized peripheral blood from AP patients (GSE194331) to validate systemic inflammatory signatures. This approach reflects the systemic cytokine milieu driving remote organ injury, though it does not directly represent pulmonary pathology.

## 3. Results

### 3.1. Identification of DEGs in AP and ALI datasets

**Fig 1:**
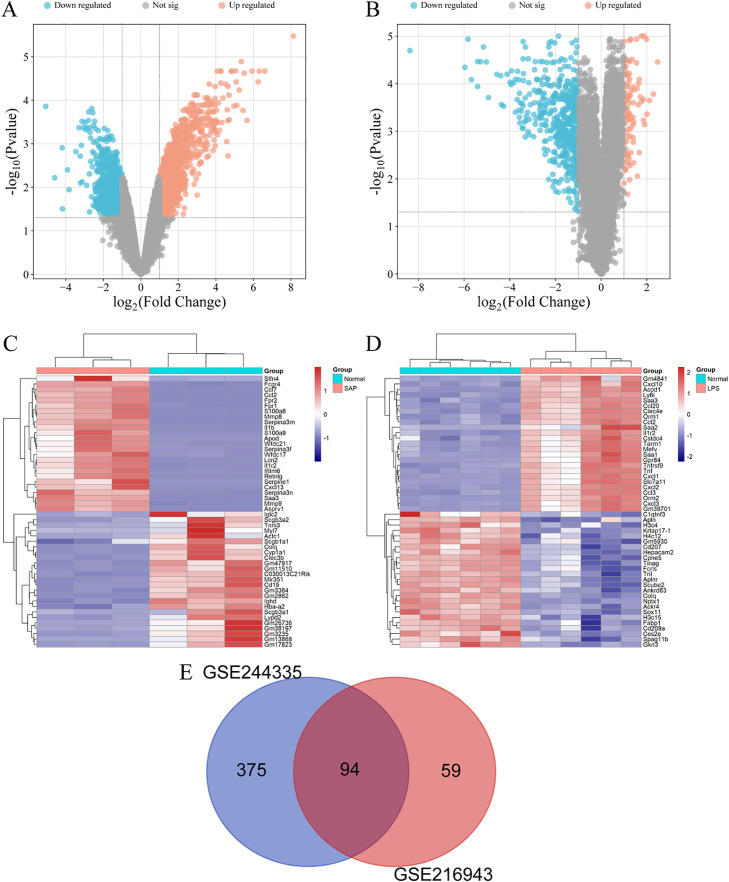
DEG identification and overlap. **(A)** Volcano plot displaying DEGs in the AP dataset. **(B)** Volcano plot displaying DEGs in the ALI dataset. **(C)** Heatmap presenting top 50 DEGs in the AP dataset. **(D)** Heatmap presenting top 50 DEGs in the ALI dataset. **(E)** Venn diagram illustrating the overlapping DEGs between the AP and ALI datasets..

The volcano plots depicting the screening process of differentially expressed genes contained in the AP dataset (GSE244335) and the ALI dataset (GSE216943), respectively, are detailed in Fig 1A and 1B. To pinpoint significant genes in the AP and ALI datasets, we employed a screening criterion where the absolute value of |log_2_FC| > 2 and the adjusted p-value < 0.05. After screening according to the aforementioned criteria, it was found that GSE244335 contained 469 significant DEGs (including 340 upregulated genes and 129 downregulated genes), while GSE216943 contained 153 significant DEGs (including 4 upregulated genes and 149 downregulated genes). The heatmaps displays the top 50 DEGs with differential expression levels in the AP and ALI datasets (Fig 1C and 1D). There are a total of 94 overlapping DEGs in the AP and ALI datasets (Fig 1E).

### 3.2. Enrichment analysis of DEGs

**Fig 2:**
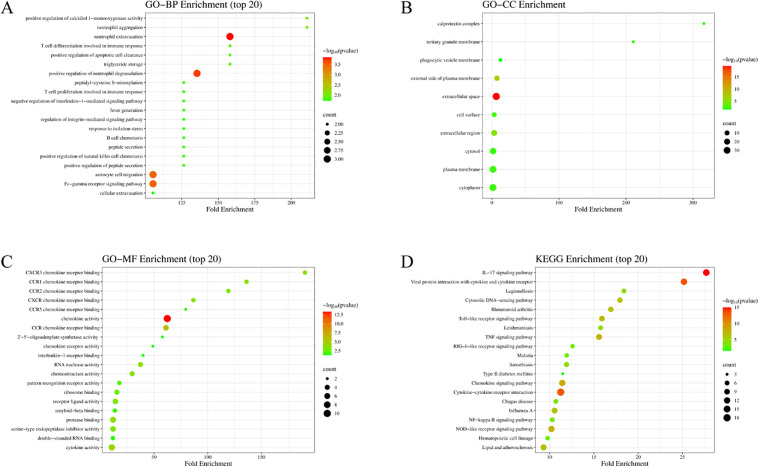
GO/KEGG enrichment of common DEGs. **(A)** Biological processes. **(B)** Cellular components. **(C)** Molecular functions. **(D)** KEGG pathways. The Y-axis represents the enriched categories, and the X-axis denotes the fold enrichment. The size of the bubbles reflects the number of genes distributed in functional regions. Note: Hierarchically related GO terms (e.g., parent/child terms for inflammatory response) are retained to show granularity levels but represent interconnected biological processes.

To investigate the biological functions of the 94 DEGs, GO and KEGG analyses were performed on them. In biological processes (BP), the DEGs are significantly enriched in interconnected immune-related processes and their regulatory networks, as well as inflammation and stress (Fig 2A). In the cellular component (CC), the DEGs are mainly involved in membrane structures, extracellular regions, and intracellular structures represented by the cytoplasm (Fig 2B). The molecular function (MF) analysis showed that the DEGs were significantly enriched in chemokine receptor binding, functions related to biological activity, receptor and binding functions, as well as functions related to enzymes and nucleic acids (Fig 2C). The KEGG pathway analysis showed that the DEGs were mainly enriched in immune signaling pathways, infectious disease – related pathways, and certain other internal medicine – associated diseases (such as rheumatoid arthritis, type 2 diabetes mellitus, and atherosclerosis, etc.) (Fig 2D). Bubble plots, generated via SRplot, were used to visualize significantly enriched terms.

### 3.3. PPI network and hub gene selection

**Fig 3:**
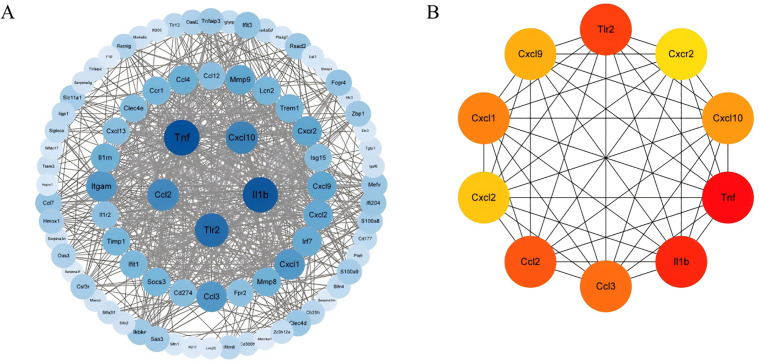
PPI network and hub genes. **(A)** PPI network of DEGs visualized via Cytoscape. **(B)** The top 10 hub genes identified from the PPI network.

The 94 DEGs were submitted to the STRING database, and the generated PPI network was visualized through Cytoscape software. This PPI network consisted of 93 nodes and 695 edges (Fig 3A). Hub genes within the network were identified via the CytoHubba plugin, and after sorting with the MCC algorithm, the top 10 selected hub genes were IL-1β, CCL3, CXCL2, CXCL10, CCL2, CXCL9, CXCL1, CXCR2, TLR2, and TNF (Fig 3B).

### 3.4. Enrichment analysis of the hub genes

**Fig 4:**
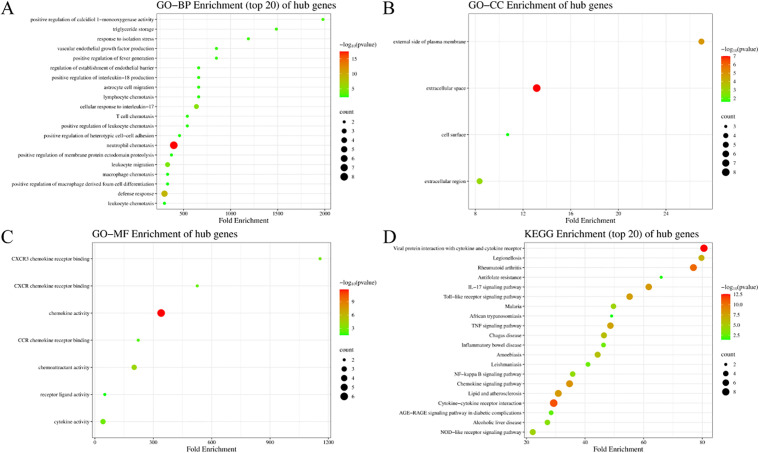
Hub gene enrichment analysis. **(A)** Biological processes. **(B)** Cellular components. **(C)** Molecular functions. **(D)** KEGG pathways. The Y-axis represents the enriched categories, and the X-axis denotes the fold enrichment. The size of the bubbles reflects the number of genes distributed in functional regions. Note: Hierarchically related GO terms (e.g., parent/child terms for inflammatory response) are retained to show granularity levels but represent interconnected biological processes.

To explore the biological roles of these 10 hub genes, GO and KEGG analyses were conducted on them. In biological processes (BP), the hub genes are significantly enriched in immune-related processes, regulation of cellular physiological activities, as well as processes related to blood vessels and metabolism (Fig 4A). In cellular components (CC), the hub genes are primarily involved in the extracellular region and cell membrane surface structures (Fig 4B). Molecular function (MF) analysis showed that the hub genes were significantly enriched in chemokine receptor binding and the functions of bioactive molecules (Fig 4C). KEGG pathway analysis indicated that the hub genes were mainly enriched in immune and inflammation-related pathways, infectious disease-related pathways, and metabolic disease-related pathways (Fig 4D). The IL-17 signaling pathway enrichment was driven by 5 hub genes: CXCL1, CXCL10, IL-1β, TNF, and CCL2, suggesting coordinated activation of neutrophil recruitment rather than isolated gene effects.

### 3.5. Verification data

**Fig 5:**
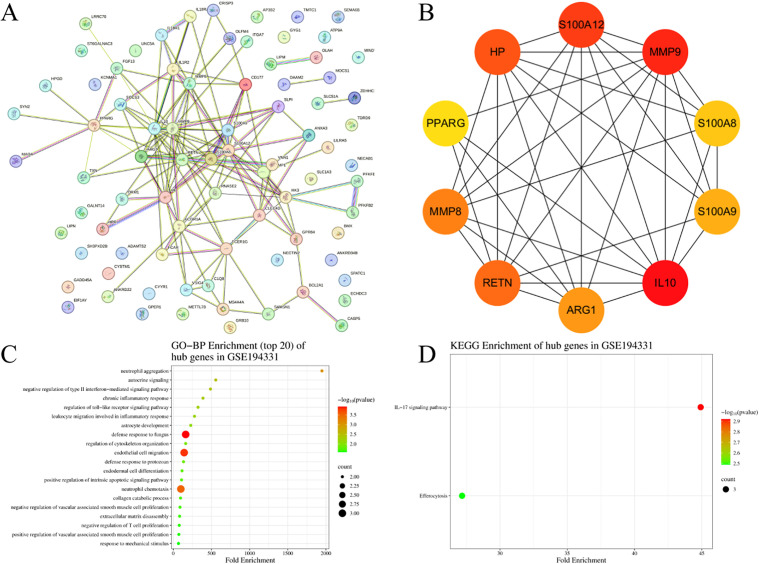
Human validation dataset results. **(A)** PPI network. **(B)** The 10 hub genes. **(C)** GO-BP enrichment analysis results of hub genes. **(D)** KEGG enrichment analysis results of hub genes.

A total of 105 DEGs were screened from the GSE194331 dataset, which were then imported into STRING to obtain a PPI network (Fig 5A). Cytoscape was used for further analysis of the PPI network, and the MCC function of the Cytohubba plugin was applied to screen out 10 hub genes, specifically including S100A9, ARG1, RETN, MMP9, HP, S100A8, MMP8, IL10, S100A12, and PPARG (Fig 5B). Subsequently, DAVID was employed to perform GO-BP and KEGG pathway analyses on these 10 hub genes from the GSE194331 dataset, and SRplot was used to generate visualized bubble plots for the GO-BP and KEGG enrichment results (Fig 5C and 5D).

**Fig 6:**
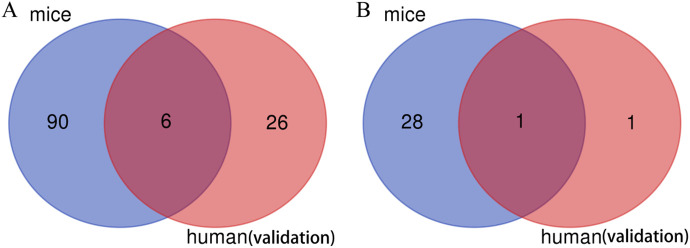
Cross-species pathway intersection. **(A)** Intersection of GO-BP enriched pathways. **(B)** Intersection of KEGG enriched pathways.

After intersecting the GO-BP pathways of hub genes from the mouse lung tissue dataset and those from the human blood dataset using a web-based Venn diagram tool, 6 common pathways were identified between the two (Fig 6A). The same method was used to identify 1 common KEGG pathway between the two (Fig 6B). The aforementioned shared pathways are presented in Table 1. These pathways primarily center on the regulation of inflammatory responses and involve the following aspects: the chemotaxis and migration of immune cells (neutrophils, leukocytes) to drive the initiation of inflammation; the positive regulation of inflammatory responses and the activity of NF-κB transcription factors to amplify inflammatory signals; the cellular response to lipopolysaccharides (pathogen-associated molecules) for recognizing infections; and the integration of inflammatory cascades via the IL-17 signaling pathway, which collectively participate in multiple stages of the occurrence and development of inflammation.

**Table 1:** Relevant parameters of the intersecting pathways.

Type	Common enrichment pathway	Mice		Human	
		Fold enrichment	Count (n)	Fold enrichment	Count (n)
GO-BP	Neutrophil chemotaxis	396.16	8	99.04	3
	Leukocyte migration involved in inflammatory response	282.97	2	278.26	2
	Positive regulation of inflammatory response	99.04	3	52.17	3
	Positive regulation of NF-κB transcription factor activity	71.31	3	48.69	3
	Inflammatory response	65.38	9	13.53	3
	Cellular response to lipopolysaccharide	58.07	6	30.43	3
KEGG	IL-17 signaling pathway	61.71	6	44.92	3

## 4. Discussion

This study systematically identified potential key genes and shared pathogenic pathways associated with acute pancreatitis-associated acute lung injury (AP-ALI), through integrated bioinformatics analysis of mouse and human datasets, providing novel insights into the molecular mechanisms underlying AP-ALI and potential targets for clinical intervention. While acknowledging etiological differences between LPS-induced direct lung injury and AP-associated indirect lung injury, both conditions converge on common terminal pathways involving neutrophil recruitment and alveolar barrier disruption [12,13]. Therefore, this cross-species analysis focuses on conserved inflammatory mechanisms rather than disease-specific pathophysiology.

PPI network analysis and hub gene screening identified the top 10 hub genes in mice, including IL-1β, CCL3, CXCL2, CXCL10, CCL2, CXCL9, CXCL1, CXCR2, TLR2, and TNF. These genes are closely associated with inflammatory regulation and immune cell recruitment, which aligns with the known pathological characteristics of AP-ALI. For instance, IL-1β, a classic pro-inflammatory cytokine, was previously shown to activate the NF-κB signalling pathway in AP, promoting the release of downstream inflammatory mediators and exacerbating pancreatic and pulmonary inflammation [14,15]. Similarly, TNF, another core pro-inflammatory factor, plays a pivotal role in the initiation and amplification of the systemic inflammatory response in SAP, and its overexpression is closely linked to the development of ALI [16,17]. The chemokines (CXCL1, CXCL2, CXCL9, CXCL10, CCL2, CCL3) and their receptor (CXCR2) among the hub genes are critical regulators of immune cell chemotaxis. CXCL1 and CXCL2, ligands of CXCR2, specifically mediate neutrophil recruitment to the site of inflammation. A previous study on LPS-induced ALI demonstrated that the CXCL1/CXCR2 axis promotes neutrophil infiltration into lung tissue, disrupting the alveolar-capillary barrier and exacerbating lung injury [18]. This is consistent with our GO enrichment analysis, which showed that DEGs are significantly enriched in ‘neutrophil chemotaxis’ and ‘leukocyte migration involved in inflammatory response’. Additionally, TLR2, a pattern recognition receptor, recognizes pancreatic damage-associated molecular patterns (DAMPs) in AP, activating downstream signalling pathways to trigger inflammatory responses (as mentioned in the Introduction), further confirming the reliability of our hub gene selection. In the human AP peripheral blood dataset (GSE194331), we identified 10 hub genes, including S100A9, ARG1, RETN, MMP9, HP, S100A8, MMP8, IL10, S100A12, and PPARG. Among these, S100A8 and S100A9 form a heterodimer that is highly expressed in inflammatory conditions and has been proposed as a potential biomarker for AP severity. Clinical studies showed that serum levels of S100A8/A9 are significantly elevated in AP patients, especially in those with severe disease, and are positively correlated with the development of organ failure [19,20]. This suggests that the hub genes identified in human datasets have potential clinical translational value, providing a basis for the development of AP-ALI diagnostic biomarkers.

Intersection analysis of GO-Biological Process (BP) pathways between mouse and human hub genes revealed 6 common pathways, including “neutrophil chemotaxis”, “leukocyte migration involved in inflammatory response”, “positive regulation of inflammatory response”, “positive regulation of NF-κB transcription factor activity”, “inflammatory response”, and “cellular response to lipopolysaccharide”. Additionally, 1 common KEGG pathway, the “IL-17 signaling pathway”, was identified. These pathways collectively reflect the core molecular mechanisms of AP-ALI, centered on inflammatory response regulation. The NF-κB signaling pathway, a key component of the common GO-BP pathways, is a central regulator of inflammatory responses. It mediates the transcription of multiple pro-inflammatory cytokines (such as IL-1β and TNF) and chemokines, playing a critical role in the progression of AP to ALI [21,22]. The ‘cellular response to lipopolysaccharide’ pathway highlights the role of pathogen-associated molecular patterns (PAMPs) in AP-ALI. In SAP, intestinal barrier dysfunction leads to bacterial translocation, and LPS (a major component of bacterial cell walls) activates immune cells via TLRs, further amplifying the inflammatory cascade and contributing to ALI [23,24]. The IL-17 signaling pathway, the only common KEGG pathway, has recently gained attention in AP-ALI research. IL-17 promotes the release of inflammatory factors and chemokines by activating downstream signaling molecules, enhancing neutrophil recruitment and lung tissue damage. A study on SAP mouse models showed that inhibition of the IL-17 signaling pathway reduces pancreatic and pulmonary inflammation, improving survival rates [25]. Our findings further confirm the importance of this pathway in AP-ALI, suggesting it may be a promising therapeutic target. While this study design identifies shared molecular signatures between AP and ALI, we acknowledge that it infers rather than directly measures the AP-ALI interface. The co-occurrence of AP and ALI may involve emergent interactions (e.g., pancreas-lung crosstalk via exosomal miRNAs or specific damage-associated molecular patterns) not captured by intersecting independent datasets. To address this, we compared our findings with published AP-ALI co-morbid studies. Notably, our identified hub genes IL-1β, CXCL2, and TNF have been independently validated in experimental AP-ALI mouse models showing concurrent pancreatic necrosis and alveolar damage, supporting their relevance to the co-morbid state despite our analytical approach [26,18]. Future transcriptomic analysis of AP-ALI combined models (e.g., cerulein-induced SAP with respiratory failure) would provide definitive validation.

A key novelty of this study is the integration of mouse and human datasets for cross-species validation. Most previous bioinformatics studies on AP-ALI have focused solely on mouse models or human samples, limiting the translational value of their findings. By comparing hub genes and pathways between mouse lung tissue (pathological site) and human peripheral blood (easily accessible clinical sample), we identified shared molecular mechanisms and potential biomarkers (such as S100A8/A9) that are applicable to both preclinical research and clinical practice. Clinically, the identified hub genes and pathways provide new directions for AP-ALI diagnosis and treatment. For example, S100A8/A9 in human peripheral blood could serve as a non-invasive biomarker for early prediction of AP-ALI development, while targeting the IL-17 signalling pathway or CXCL1/CXCR2 axis may offer new therapeutic strategies. Additionally, the NF-κB pathway, as a central node of inflammatory regulation, could be targeted to inhibit the excessive inflammatory response in AP-ALI, reducing organ damage. We acknowledge a critical limitation in our cross-species validation: the human dataset (GSE194331) represents peripheral blood rather than lung tissue. Therefore, our validation supports overlapping systemic inflammatory responses between AP and ALI mouse models, rather than direct validation of pulmonary pathology. However, this design offers clinical relevance: (a) peripheral blood is the primary sample type available for AP patient monitoring; (b) circulating inflammatory mediators (e.g., S100A8/A9, IL-6) drive remote lung injury; (c) S100A8/A9 identified in our human blood analysis has been previously detected in AP-ALI lung tissue by immunohistochemistry [27]. Future validation should prioritize human AP-ALI lung tissue (e.g., from autopsy studies) or bronchoalveolar lavage fluid to directly confirm pulmonary expression patterns.

Despite its contributions, this study has limitations. The small sample sizes of the mouse datasets may introduce bias in DEG and hub gene selection. Specifically, the small sample sizes in mouse datasets (n = 3 for GSE244335) may limit statistical power, with an estimated FDR of 15%−20% for marginal DEGs. However, the high effect sizes of identified hub genes support their reliability. Additionally, the findings lack experimental validation through wet-lab methods such as qPCR, Western blot, or functional assays. The mouse models used (e.g., cerulein- or LPS-induced) do not fully replicate human AP-ALI pathology, which commonly arises from gallstones or alcohol, suggesting possible mechanistic differences [28,29]. Furthermore, the human data derived from peripheral blood (GSE194331) lack validation in lung tissues—the primary site of injury in ALI. Further studies with larger samples, relevant animal models, and human lung tissue are warranted. Moreover, our analysis intersected separate AP and ALI datasets rather than analyzing AP-ALI co-occurrence. While this identifies shared inflammatory mechanisms, it may miss interaction-specific pathways. The lack of publicly available AP-ALI combined transcriptomic datasets with adequate sample sizes currently prevents direct validation, though our hub genes overlap with mechanistic reports from AP-ALI animal models. We acknowledge that centrality-based hub selection (MCC) prioritizes highly connected inflammatory mediators that are well-represented in protein interaction databases. While this approach identifies robust network nodes, hub status reflects topological connectivity rather than validated pathogenic importance. Therefore, we interpret these genes as candidate markers requiring functional validation (e.g., genetic knockout or neutralizing antibody studies) to confirm causal involvement in AP-ALI.

Future research should prioritise experimental validation of key hub genes such as IL-1β, CXCL2, and S100A9 using relevant cellular and animal models of AP-ALI, including assessing the therapeutic potential of agents like CXCR2 inhibitors. Studies should also incorporate larger and more clinically diverse human cohorts—encompassing multiple etiologies and paired blood and lung tissue samples—to enhance the translational robustness of biomarker signatures. Furthermore, mechanistic investigation into cross-pathway interactions, particularly between IL-17 and NF-κB signalling, is essential to elucidate central regulatory mechanisms. These efforts should culminate in the development and preclinical evaluation of targeted therapies, such as small-molecule inhibitors or neutralizing antibodies against these core pathways.

## 5. Conclusion

In summary, this study identified 94 common DEGs and 10 hub genes in mouse AP-ALI models, as well as 10 hub genes in human AP peripheral blood samples. Cross-species validation revealed 6 common GO-BP pathways and 1 common KEGG pathway (IL-17 signaling pathway) associated with AP-ALI. These findings highlight shared mechanisms between systemic inflammation (human blood) and lung injury (mouse models), suggesting that circulating inflammatory mediators may drive AP-ALI pathogenesis and offering accessible biomarkers (e.g., S100A8/A9) for early identification of lung injury risk in AP patients. However, as peripheral blood reflects systemic inflammation rather than direct pulmonary pathology, these signatures require validation in human lung tissue or Bronchoalveolar lavage fluid to confirm local pathogenic relevance.

## Supporting information

S1 File GSE244335-DEGs. Analysis results of DEGs in GSE244335.
(CSV)

S2 File GSE216943-DEGs. Analysis results of DEGs in GSE216943.
(CSV)

S3 File GO-BP. GO-BP enrichment results of 10 hub genes.
(TXT)

S4 File GO-CC. GO-CC enrichment results of 10 hub genes.
(TXT)

S5 File GO-MF. GO-MF enrichment results of 10 hub genes.
(TXT)

S6 File KEGG. KEGG enrichment results of 10 hub genes.
(TXT)

S7 File R code. R code used during DEGs analysis.
(TXT)
